# India's Vital Statistics

**Published:** 1921-04-02

**Authors:** 


### India's Vital Statistics.

According to an abstract of vital statistics for British India for the first quarter of last year, issued in mail week, Delhi City suffered about 1,000 less deaths than in the preceding quarter, the difference in the birth-rate also being about the same.

Bihar and Orissa figures show that deaths exceeded births by 21,138 in the towns, and by 20,937 in the districts. Births in the towns of the United Provinces increased by more than 3,000, deaths being 261 less than births. Deaths in the districts, however, exceeded the number of births by 19,847. In the Punjab births and deaths in seventeen towns were both about 1,000 less, and in districts also both these features prevailed almost to a same degree. About 4,000 less births were recorded in the towns of Madras, several districts showing an increase of more than 50,000. Deaths were less in the towns by 2,000.

The birth-rate in Bombay was lower by nearly one-third, both in the towns and districts, whereas the death-rate increased all round by 10 per cent.

The birth-rate in Burma showed a decrease in the towns by more than 10 per cent., and in the districts also there was a decline of about 3,000. Deaths in the towns, however, increased by 2,000, an almost corresponding increase being noticeable in the districts.

